# Financial Decision Making and Survey Responses of Older Couples

**DOI:** 10.1093/geroni/igaa057.995

**Published:** 2020-12-16

**Authors:** Angela Curl, Estevan Tobias Molina

**Affiliations:** Miami University, Oxford, Ohio, United States

## Abstract

The Health and Retirement Study (HRS) asks mid-life and older couples to identify the person most knowledgeable about household finances to provide responses to financial questions (“financial respondent” [FR]). The purpose of this study is to analyze predictors of a female FR, with a focus on gendered financial decision making. This study analyzed HRS data from 2012 to 2018. Our sample consisted of 5,038 married/partnered couples over the age of 50 (at baseline), after eliminating cases with missing data on study variables. Using HLM 6.08 software, we computed the odds of couple households having a female FR. Our main predictor was the amount of female financial decision making power, as measured using eight Likert scale items (0=male always makes this decision, 4=female always makes this decision), for purchases (e.g., cars, major appliances) and financial activities (e.g., paying bills, buying groceries); summed into a scale (range: 0-32). Our models controlled for race, ethnicity, education, income, age, health, cognitive ability, and depressive symptoms. Results indicate that men were more likely to be the FR, but when the female makes more household financial decisions then she is more likely to be the FR (OR = 1.16, p<.01). Black race, lower income, and time also increased the odds of a female FR. This study highlights women’s historical and ongoing limited power in financial matters of married couples. Financial knowledge and decision making skills should be encouraged for both partners in couple households.

